# Complicated Pneumonia in a Child: Hydropneumothorax Associated with MIS-C and GAS Superinfection

**DOI:** 10.3390/pediatric16040071

**Published:** 2024-09-30

**Authors:** Snezhina Lazova, Nadzhie Gorelyova-Stefanova, Yoanna Slabakova, Iren Tzotcheva, Elena Ilieva, Dimitrina Miteva, Tsvetelina Velikova

**Affiliations:** 1 Pediatric Clinic, University Hospital “N. I. Pirogov”, 21 “General Eduard I. Totleben” Blvd., 1606 Sofia, Bulgaria; nadzhie.gorelyova@gmail.com (N.G.-S.); irenmd@yahoo.com (I.T.); 2 Department of Healthcare, Faculty of Public Health “Prof. Tsekomir Vodenicharov, MD, DSc”, Medical University of Sofia, Bialo more 8 Str., 1527 Sofia, Bulgaria; 3 Medical Faculty, Sofia University St. Kliment Ohridski, 1 Kozyak Str., 1407 Sofia, Bulgaria; tsvelikova@medfac.mu-sofia.bg; 4 Specialized Hospital for Active Treatment of Infectious and Parasitic Diseases “Prof. Ivan Kirov”, Bulgaria Blvd. “Akademik Ivan Evstratiev Geshov” 17, 1431 Sofia, Bulgaria; y.slabakova@abv.bg; 5 Department of Diagnostic Imaging, University Emergency Hospital (UMHATEM) “N. I. Pirogov”, Bul. “General Eduard I. Totleben” 21, 1606 Sofia, Bulgaria; ilievaelena@abv.bg; 6 Department of Genetics, Faculty of Biology, Sofia University St. Kliment Ohridski, 8 Dragan Tzankov Str., 1164 Sofia, Bulgaria; d.georgieva@biofac.uni-sofia.bg

**Keywords:** case report, hydropneumothorax, community-acquired pneumonia, complicated pneumonia, MIS-C, GAS superinfection

## Abstract

A hydropneumothorax is an uncommon complication of pneumonia, particularly in pediatric patients, and typically arises secondary to conditions such as malignancies, esophageal-pleural fistula, thoracic trauma, or thoracocentesis. While pneumothorax is rarely reported in adults with COVID-19 and is even less common in children, isolated cases have been noted in those with Multisystem Inflammatory Syndrome in Children (MIS-C). A recent alert has also been issued about increased Group A Streptococcus (GAS) infections in Europe. Against this background, the primary aim of this case report is to describe a rare and severe complication of pneumonia in a previously healthy child with MIS-C and a positive throat culture for GAS.

## 1. Introduction

Community-acquired pneumonia (CAP) is a common pediatric infection and, by definition, is “the presence of signs and symptoms of pneumonia in a previously healthy child due to an infection which has been acquired outside hospital” [1,2].

Most CAP cases in children do not require hospitalization [3,4]. However, the complications should not be underestimated. A British Thoracic Society Paediatric Pneumonia Audit in 2011 concluded a complication rate of 7.1% from 2200 cases in 77 hospitals, with empyema assessed in 4.4% and lung abscess in 0.9% of the children [5]. Risk factors for developing CAP include immunodeficiencies, chronic lung diseases, cystic congenital thoracic malformations, and inhaled foreign bodies [6].

A hydropneumothorax is a rarely reported complication of pneumonia, especially in the pediatric population [7,8]. It is defined as the presence of both free fluid and air within the pleural space. Usually, hydropneumothorax occurs secondary to various conditions such as malignancies, esophageal-pleural fistula, thoracic trauma, and thoracocentesis [9]. Currently, several case series reported a low incidence of pneumothorax in adults with COVID-19, and it is even rarer in children. There have been reports of children with MIS-C developing necrotizing pneumonia and pneumothorax, and some studies suggest that GAS may be involved [10]. GAS is a type of bacteria that can cause various infections, from a mild sore throat to severe invasive diseases such as necrotizing fasciitis. GAS can also cause pneumonia and, in some cases, can lead to necrotizing pneumonia. GAS, by itself, could be associated with post-infectious inflammatory reactions (PIIRs) [11].

Since these complications are uncommon, we aimed to present a rare severe pneumonia complication in a previously healthy child with MIS-C and positive throat culture for GAS. We also discussed the differential diagnosis, possible risk factors, management, and prognosis of the patient.

For the period December 2020–February 2022, 51 patients meeting the MIS-C criteria were admitted to the pediatric department of UMHATEM “N. I. Pirogov” [12,13,14,15]. Since then, only one highly suspected MIS-C has been observed and is described in the following case report.

## 2. Case Report

We present a case of a 2-year-old previously healthy boy with a complete immunization schedule and a history of data for asymptomatic COVID−19 infection (November 2020) and acute upper respiratory tract infection a month before the present condition.

**Figure 1:**
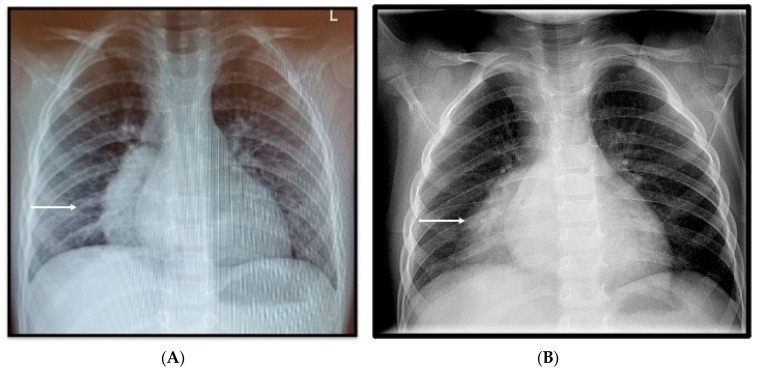
Frontal chest X-ray (**A**). Initial outpatient X-ray performed at another medical facility, a day prior to admission, reveals large dense paracardial opacity (white arrow); (**B**). Day 1 of hospital admission, showing inhomogeneous right para- and retrocardiac ill-defined dense opacity and air bronchogram (white arrow).

On the 10th of April 2022, the child presented in the pediatric emergency room with symptoms of unproductive cough, abdominal pain, and fever of up to 40.2 °C during the past six days and radiologic data for right-sided pneumonia (Figure 1A). The day before hospitalization, he had severe back pain, reduced fluid and food intake, and diuresis.

At the admission to the Pediatric ward, the child was intoxicated, pale, febrile, without signs of decompensation—transcutaneous oxygen saturation—94–95%, and exhibited tachycardia (143/min) and tachypnea (38/min). On chest auscultation, diminished vesicular breathing in the right parasternal region was observed, and the peripheral lymph nodes were not enlarged.

Laboratory tests on admission revealed hyperinflammation with elevated CRP—300.0 mg/L (<5), PCT—37.98 ng/mL (n < 0.5), LDH 558 U/l (<248), and leukocytosis 14.28 × 10^9^/L. The laboratory research dynamics are presented in Table 1 and Table 2. The chest X-ray did not show a significant dynamic compared to the previous one, performed in the outpatient setting (Figure 1B), with abdominal sonography data for minimal ascites.

**Figure 2:**
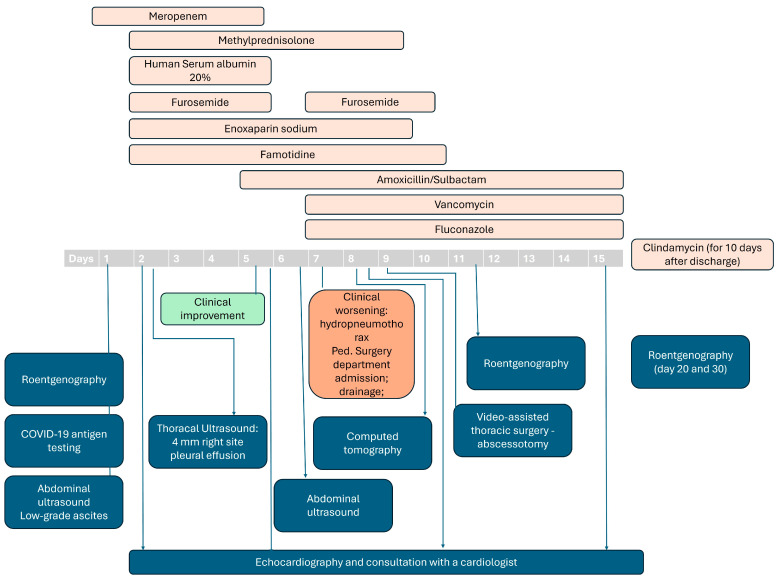
Therapeutic plan during the hospital stay.

Initially, the child was evaluated as having infiltrative pneumonia, and empiric broad-spectrum antibiotic therapy with meropenem was initiated. On the second day of hospitalization, the child’s condition worsened with periorbital edema, moderate edema of the extremities, tachypnea with grunting, and fatigue. We observed higher values of the acute-phase markers of inflammation—ferritin, LDH, and D-Dimer. The initial throat culture was positive for GAS. Echocardiography (Echo) revealed evidence of pericardial reaction (thickened (up to 4.0 mm) hyperechoic pericardium) and small right-side pleural effusion, as well as intact atrial septum, in a neutral position, and possible slight dilatation of the LV—index LV/Ao 1.63, despite hypokinetic areas (anteroseptal, basal, and middle sections) with preserved systolic ventricular ejection fraction. On ECG monitoring, there were no signs of pericarditis. Unfortunately, cardiac indices (usually available in our department—CPK-MB and troponin I) were not assessed. However, there was a tendency for a decrease in serum albumin, highly elevated IL-6 (547 pg/mL, reference range of below 6 pg/mL), and positivity for anti-SARS-CoV-2 IgG (548 BAU/mL, ref. range below 22). The ECHO examination led to a discussion of possible MIS-C syndrome with pulmonary and cardiac involvement. Therefore, systemic corticosteroids, human serum albumin 20%, furosemide, and low-molecular-weight heparin (enoxaparin sodium) were added to the treatment plan (Figure 2).

**Figure 3:**
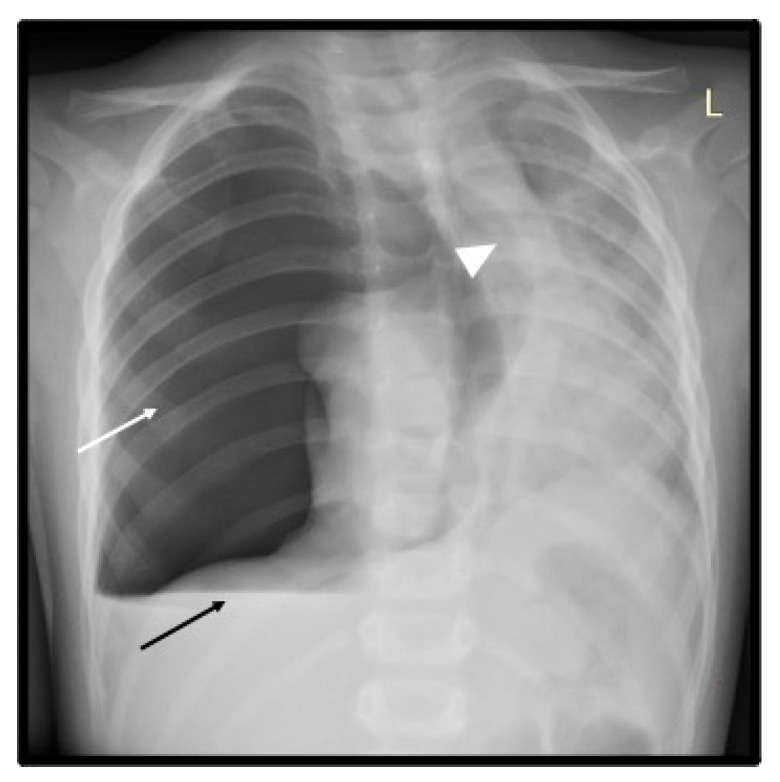
Day 7 of hospital admission. Frontal chest X-ray reveals a large right pneumothorax (white arrow) with a marked shift of the mediastinum to the left (white arrowhead) as demonstrated by the leftward deviation of the trachea and cardiac silhouette and small hydrothorax with air-fluid level (black arrow).

On the third day of the hospital stay, mild to moderate edema of the limbs and periorbital edema persisted with diminished urinary output (diuresis 1.6 mL/kg/h), and there was an erythematous rash on the face—cheeks and cheekbones and gradually appearing palmar erythema. Over the next three days, there was a significant improvement in the general condition, and the child remained afebrile. On the seventh day of hospitalization, the child had a good tonus, reduced cough, and a normal appearance. Suddenly, without any objective predisposing factors, the child presented with deterioration of the general condition, was exceptionally restless, tachypneic (40/min), dyspneic with supraclavicular retractions, complained of abdominal pain, and had severely low-pitched breathing throughout the right chest with tympanic percutaneous tone on the same side. The vital signs remained stable—SpO2 of 93%, HR 131/min, and blood pressure 131/90 mmHg. The chest X-ray showed total pneumothorax with a fluid level at the base, a shift of the mediastinum to the left (tension pneumothorax), and hypoventilation of the parenchyma on the left (Figure 3).

**Figure 4:**
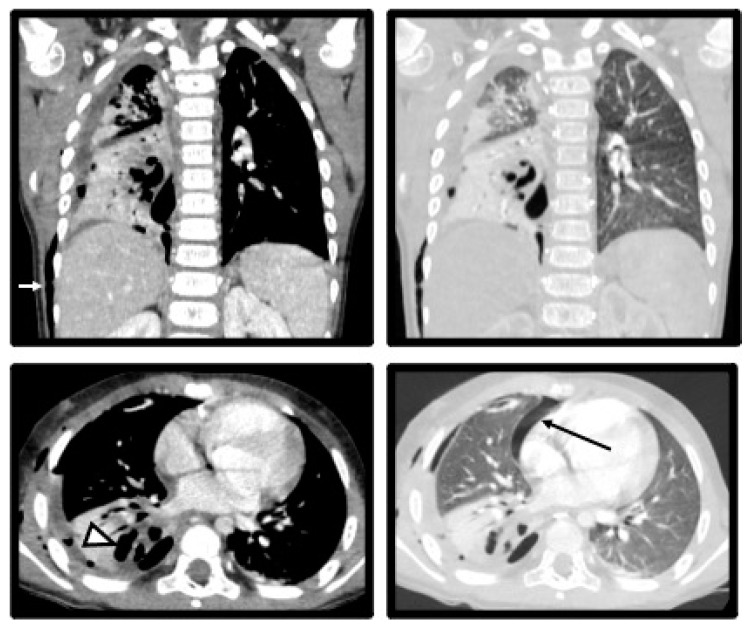
Day 7 of hospital admission. Chest CT (coronal and axial MPRs, mediastinal and lung windows) post-thoracocentesis reveals small residual pneumothorax (white arrow), pleural empyema with well-defined area of non-enhancing lung with cavitation adjacent to empyema (arrowhead) and subcutaneous emphysema (black arrow).

The child was referred to the pediatric surgery department for an emergency drainage procedure. Thoracentesis was performed with air and cloudy exudate evacuated. The chest CT scan post-thoracocentesis revealed a small residual pneumothorax, pleural empyema with a well-defined area of the non-enhancing lung with cavitation adjacent to empyema, and subcutaneous emphysema (Figure 4). In the following days, 50–80 mL of hemorrhagic-serous fluid was drained. The follow-up echocardiography showed a tendency for gradual improvement without aneurismal signs.

**Figure 5:**
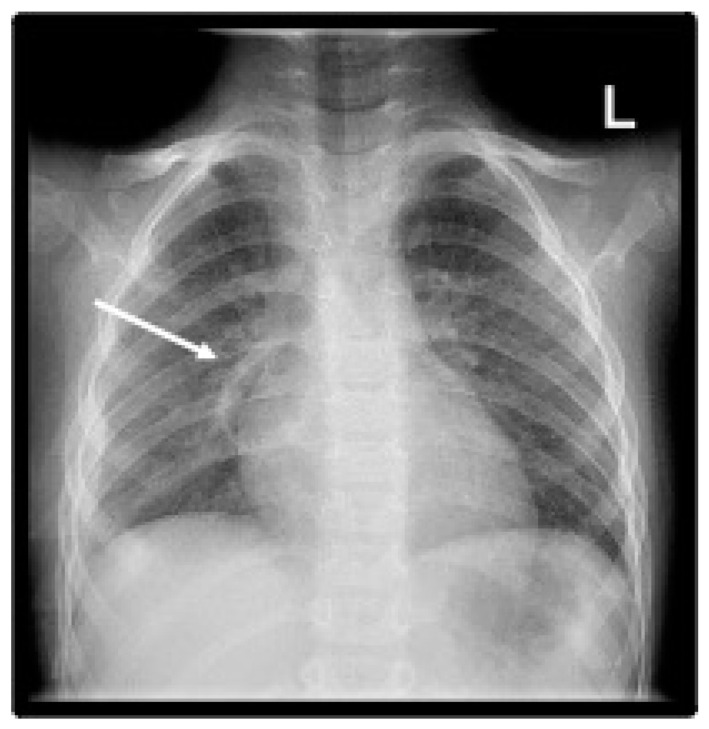
Day 20 of hospital admission. Frontal chest X-ray reveals right paracardiac thin-walled postpneumonic pneumatocele (white arrow).

**Figure 6:**
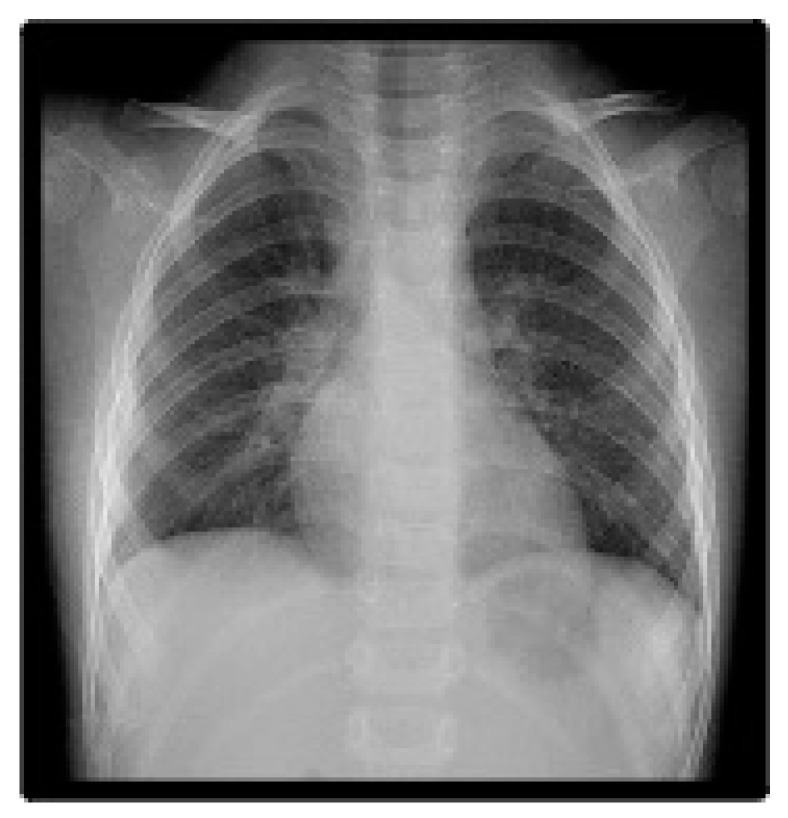
Follow-up frontal chest X-ray (20 days after hospital discharge) demonstrates full recovery.

Two days after thoracocentesis, the patient required video-assisted thoracoscopic surgery (VATS), which was indicated by the developed additional complications—empyema and lung abscess. An abscess was found in the 10th lung segment, followed by an abscessotomy. Blood culture, feces, stool culture, pleural fluid culture, and abscess material were negative. The child’s condition gradually improved; the drains were removed, and the inflammatory indices normalized. On the 15th day, the patient was discharged with ten-day peroral therapy with Clindamycin. On the 20th day, a frontal chest X-ray revealed a right-side paracardiac thin-walled postpneumonic pneumatocele (Figure 5). A month later, the child was followed up with a normalized chest X-ray and normalized echocardiography without any signs of residual complications (Figure 6).

## 3. Discussion

Worldwide, only a few cases of hydropneumothorax have been reported. Compared to our patient, the condition usually manifests at the beginning of the disease course. Furthermore, the children typically have a medical history of malignancies and neonatal prematurity [8,9]. As an example, in 2016, a 5-month-old baby with hydropneumothorax presented in the emergency department 3 days after the onset of symptoms and complicated history in the neonatal period—born from a twin pregnancy in the 28th gestational week with surfactant application and an unsatisfactory weight gain after discharging [9].

On the contrary, our patient was a full-term born with normal development and no comorbidities. One month before the hospitalization, he had an upper respiratory tract infection, and he had an asymptomatic SARS-CoV-2 infection a year and a half ago. At admission and during the first days of the hospital stay, the child’s condition was stable but impaired with remarkably high inflammatory markers (C-reactive protein, procalcitonin, interleukin—6, ferritin, D-dimer), with hypoalbuminemia, peripheral edemas, and skin rash. Due to the epidemic situation at the time (second pandemic year), multisystem involvement and positive serology for SARS-CoV-2 RBD-specific IgG antibodies, MIS-C syndrome was considered as a possible diagnosis. In our case, there was clear evidence for epidemiological and immune contact with SARS-CoV-2—the child had laboratory-proven acute asymptomatic SARS-CoV-2 infection a year and a half ago, history data for acute respiratory infection four weeks ago, and high titers of anti-SARS-CoV-2 IgG antibodies. 

However, the throat culture at admission was positive for beta-hemolytic streptococcus (group A streptococcus, GAS) with negative hemocultures. The chest radiology did not show typical imaging characteristics for acute COVID-19 or MIS-C. Moreover, it is consistent with lung infiltrate evolving in necrotizing pneumonia and spontaneous unilateral pneumothorax typically associated with a severe bacterial infection. On the other hand, the child’s hemodynamics were stable without the features of toxic shock syndrome related to toxin-producing bacteria. Other possible diagnoses were PIIR or atypical Kawasaki disease. According to the study by Abraham et al., PIIR was associated with pleuropneumonia, hospitalization in an intensive care unit (ICU), and elevated C-reactive protein (CRP) [11]. It occurs after a median of 8 days (5–12) and presents with fever, arthritis, and pleural effusion. In our patient, the deterioration appeared on the seventh day. Still, the skin rash, abdominal pain, pleural effusion, ascites, and pericardial reaction were evident early in the clinical course preceding the parapneumonic complications. The criteria for Kawasaki disease were also not met.

A remarkable improvement was observed throughout therapy during the first 7 days after the hospitalization. With a sudden deterioration in the clinical condition, the patient presented with hydropneumothorax. It is an unusual case as to the late manifestation of the complication and the clinical improvement of the child beforehand. 

As stated, hydropneumothorax is generally a secondary complication of community-acquired pneumonia. One of the latter is necrotizing pneumonia, commonly associated with the presence of pneumatocoeles. The risk of pneumothorax is excessive if it occurs towards the parenchyma border. Another severe complication of necrotizing pneumonia is a bronchopleural fistula, the presence of which results in pneumothorax or pyopneumothorax [3].

One case reported by Montgomery and Finckwith describes hemopneumothorax in 17-year-old adolescents with COVID-19. The authors suspected spontaneous rupture from a bleb induced by the coagulopathic state associated with the intercurrent SARS-CoV-2 infection [16]. Hemopneumothorax was described in the literature in a 23-year-old male with no reported past medical history and current COVID-19 due to vascularized bullae rupture [17]. Multiple case reports and case series were published with a low incidence of pneumothorax in COVID-19 [18]. Moreover, pneumothorax was described in less than 1% of autopsy material of individuals with COVID-19 [19].

A review of the CT features confirms the low incidence of pneumothorax as a feature of COVID-19 [20]. In some cases, SP was described as a late complication even weeks after recovery from COVID-19 infection [21].

Pediatric reports of COVID-19 infection and pneumothorax are also rare. Hashemi describes a case of a 2-year-old boy with hyper IgM syndrome and COVID-19 infection. [22] A case with MIS-C and spontaneous pneumothorax was reported by Amir Saeed et al. in a 5-year-old boy. Laaribi et al. described two boys with COVID-19 (a 9-month-old and an 18-month-old) who developed bilateral spontaneous pneumothorax before admission [23]. The cases are reported as acute COVID-19. However, the reported characteristics are consistent with MIS-C.

Similar to our case, Revenco et al. described a 13-year-old boy with COVID-19 who presented with hemoptysis, symptoms of MIS-C associated with COVID-19, and bacterial superinfection evolving into necrotizing pneumonia and spontaneous pneumothorax [24]. Sputum bacteriology showed the presence of Str. viridans, St. aureus, and respiratory panel (PCR), and the existence of Enterovirus and Rhinovirus. During the hospital stay, a second sputum culture showed the presence of Cryptococcus, indicating viral, bacterial, and fungal superinfection. In our case, only classic microbiology assays were performed: throat smear, hemocultures, and thoracic fluid aspirate. The isolated GAS could be the possible causative agent of bacterial superinfection. There have been reports of children with MIS-C developing necrotizing pneumonia and pneumothorax, and some studies suggest that GAS may be involved in these cases. GAS is a type of bacteria that can cause various infections, from a mild sore throat to severe invasive diseases such as necrotizing fasciitis. GAS can also cause pneumonia, and in some cases, this can lead to necrotizing pneumonia. Our patient showed signs of complicated bacterial superinfection after 13 days of illness and 7 days of hospital treatment. However, the mechanisms of bacterial–viral–fungal–SARS-CoV-2 coinfection and interaction require further study.

It is also possible to have co-existing mild temporary inappropriate secretion of ADH in the context of the hyper-inflammatory state and severe complicated pneumonia. The blood sodium level remained borderline low during hospitalization—at admission, 133.5 mmol/L; day 5, 137 mmol/L; day 7, 137; day 9, 134, with urine specific gravity-SG as follows—1.015, 1020. On the other hand, there is an initially low albumin level, which also could play a role in water retention and decreased diuresis. Regardless of the etiologic cause, fluid retention is controlled during treatment.

However, as the condition was initially categorized as a pulmonary infiltrate, together with the stable hemodynamics (without clinical symptoms of heart failure and/or myocarditis), unfortunately, we did not assess the cardiac indices (usually available in our department—CPK-MB and troponin I). After the evolution of the skin rash, edema, and increasing inflammatory markers, the MIS-C diagnosis was discussed, and the condition was followed up with ECHO examination. On the other hand, the clinical and laboratory constellation did not met the MIS-C case definition, and diagnosis of MIS-C remained suspected.

This case was the last suspected MIS-C in our department, contrasting with 51 MIS-C cases for the previous period (December 2020–February 2021). As observed in other big centers and highlighted in a recently published study, there is a significant global decline in MIS-C incidence [25]. The authors discuss the role of COVID-19 vaccination. In contrast, there is low vaccine coverage among children in Bulgaria and broad vaccine skepticism.

However, the throat culture at admission was positive for beta-hemolytic streptococcus (group A streptococcus, GAS) with negative hemocultures. While Invasive group A streptococcus (iGAS) is still a sporadic infection, notifications are slightly higher than expected at this time of year and remain relatively high in children compared to what we typically see: 701 iGAS cases in children aged 18 years and under, compared to 205 cases in the 2017 to 2018 season. Sadly, so far this season, there have been 401 deaths (from any cause recorded within 7 days of an iGAS infection diagnosis) across all age groups in England. This statistic includes 47 children aged under 18 years in England [26,27,28].

We can speculate that scarlet fever could be mentioned in the differential diagnosis. However, the skin rash was not typical, and the child’s age group was not the most affected, according to the literature data (5 to 15 years old) [29]. Furthermore, the patient could be a carrier (GABHS carriage), especially when the drainage was clear/not contaminated. By definition, Streptococcus pyogenes carriers are patients who exhibit no symptoms, although the throat swab culture or rapid strep test is positive. Consecutive serologic testing of those patients shows no elevation in antibody levels. Although primary schoolers are the most common carriers, we cannot exclude the possibility [30]. Sputum is known to be the best specimen for the etiology of pneumonia. Unfortunately, this is technically difficult in young children. Additionally, a pharyngeal smear does not always indicate the etiology of lower respiratory tract infections. Nevertheless, the positive throat culture could not be neglected if the pulmonary infiltrate evolved into necrotizing pneumonia with negative cultures of all assessed specimens. 

In the last two years, invasive streptococcal disease and scarlet fever have notably increased. The condition affects mostly preschoolers. According to data from the World Health Organization, an increase in the death rate was also noted in many European countries in 2022, such as the United Kingdom, France, Ireland, the Netherlands, and Sweden [31]. The CDC noted similar reports with case inflation in the USA. Invasive group A streptococcal disease, if defined by life-threatening complications with a positive culture from a normally sterile site, could be clinically associated with pneumonia-like conditions in the described case [32].

Furthermore, one could speculate that the case describes pneumonia and abscesses, hydropneumothorax, and empyema as complications of COVID-19 and GAS superinfection developed in invasive streptococcal disease. The patient was monitored and followed up for two years—no renal complications, such as glomerulonephritis, were observed.

A similar case report was published on preschoolers presenting with purulent cardiological complications and splenic involvement [33] and potentially lethal necrotizing pneumonia after sepsis caused by invasive GAS [34]. In a 2-year-old child from Guatemala with pneumonia and empyema caused by invasive GAS, antibiotics and thoracentesis were insufficient for resolution of symptoms. IVID and aspirin were used due to high clinical suspicion of Kawasaki disease [35]. Although invasive GAS infection is most common in children, case reports of adults with community-acquired pneumonia have been published in recent years [36].

The vast majority of the MIS-C cases in our department occurred from December 2020 to December 2021, except for two (January and February 2022). It is important to note that these data may not represent the entire country, as there is no national MIS-C registry. However, it does provide insight into the trend of MIS-C hospital admissions in the main emergency hospital in the capital city. During this time, there was a shift in the epidemiology of pediatric infectious diseases, characterized by a surge in GAS and iGAS cases, an increase in Varicella cases, and a high incidence of complicated and non-complicated infiltrative cases of pneumonia, B. pertussis, and Mycoplasma pneumonia infections. The case in question meets the diagnostic criteria for MIS-C. Nevertheless, the atypical pulmonary manifestation of COVID-19 and MIS-C, as well as the presence of GAS isolate in the throat smear, remain open for discussion.

## 4. Conclusions

In conclusion, bacterial community-acquired pneumonia and its possible late complications are not to be underestimated in a pediatric patient, disregarding the comorbidities and medical history. In the context of COVID-19, MIS-C, the warning about the rise in GAS infections, and possible GAS-associated post-infectious inflammatory reactions (PIIRs), all pediatric cases with hyperinflammatory state should be evaluated completely with broad differential diagnosis.

## Figures and Tables

**Table 1:** Laboratory evaluation of the patient during the 15-day hospital stay.

	Day 1 *	Day 2	Day 5	Day 7 **	Day 8	Day 9	Day 10	Day 15
**WBC/Leukocytes, 10^9^/L**	14.28	N/A	23.24	23.3	30.0	35.8	201	8.4
**Neutrophils abs, 10^9^/L**	11.23	N/A	14.78	17.5	22.9	32.4	1.81	3.5
**Neutrophils, %**	78.7	N/A	63.3	74.9	76	92.1	73	41
**Lymphocytes, abs**	1.99	N/A	5.6	4.3	5.4	2.4	4	4.3
**Lymphocytes, %**	13.9	N/A	24.1	18.3	18.2	6.9	20	56
**PLT, 10^9^/L**	165	N/A	615	488	623	638	791	627
**Hgb, g/L**	121	N/A	122	97	N/A	N/A	111	95
**ESR, mm/h**	23	N/A	20	N/A	N/A	N/A	N/A	N/A
**CRP, mg/L**	300	312.5	5.18	208.8	126.9	N/A	188.3	17.6
**Procalcitonin, ng/mL**	37.98	N/A	2.05	1.08	N/A	N/A	N/A	N/A
**Fibrinogen, g/L**	N/A	N/A	N/A	3.3	N/A	N/A	6.2	N/A
**D-Dimer, mg/L**	236	1125.0	N/A	644.0	N/A	N/A	N/A	N/A
**ASAT, U/L**	N/A	N/A	N/A	20	N/A	N/A	22	N/A
**ALAT, U/L**	N/A	N/A	N/A	6	N/A	N/A	8	N/A
**Albumin, g/L**	37	33	N/A	38	N/A	N/A	37	N/A
**Total protein, g/L**	61	56	N/A	59	N/A	N/A	65	N/A
**LDH, U/L**	558	277	N/A	N/A	N/A	N/A	N/A	N/A
**Ferritin, ng/mL**	251	267	N/A	N/A	N/A	N/A	N/A	N/A
**Total protein g/L**	61	56	73	59	N/A	N/A	65	N/A
**IL-6**	N/A	547	N/A	N/A	N/A	N/A	N/A	N/A

* at admission, ** spontaneous hydropneumothorax; WBC PLT Hgb ESR CRP ASAT ALAT LDH. N/A—not available.

**Table 2:** Peak and nadir in selected laboratory values during the hospital stay. N/A—not available.

	Peak 1	Peak 2	Nadir 1	Nadir 2	Reference Value
**WBC/Leucocytes 10^9^/L**	23.24	35.8	14.28	8.4	4.1–11.0 G/L
**PLT 10^9^/L**	615	791	165	627	140–440 G/L
**HBC g/L**	122	121	97	95	120–160 g/L
**CRP mg/L**	312.5	208.8	51.8	17.6	<0.50 mg/dL
**Procalcitonin ng/mL**	37.98	1.08	N/A	N/A	<0.50 ng/mL
**Fibrinogen g/L**	3.3	6.2	N/A	N/A	1.5–4.5 g/L
**D-Dimer mg/L**	1125.0	644.0	236	N/A	500
**ASAT**	20	N/A	22	20	20–60 U/L
**ALAT**	6	N/A	8	6	5–45 U/L
**Albumin**	37	47	33	37	34–42 g/dL
**Total protein**	61	73	56	59	61–79 g/L
**LDH U/L**	558	N/A	277	N/A	150–500 U/L
**Ferritin**	267	N/A	251	N/A	10–60 mg/L
**IL-6**	547	N/A	N/A	N/A	0–6.4 pg/mL

## Data Availability

The data presented in this study are available on request from the corresponding author.
